# Direct imaging of changes in aerosol particle viscosity upon hydration and chemical aging†
†Electronic supplementary information (ESI) available. See DOI: 10.1039/c5sc02959g


**DOI:** 10.1039/c5sc02959g

**Published:** 2015-11-12

**Authors:** N. A. Hosny, C. Fitzgerald, A. Vyšniauskas, A. Athanasiadis, T. Berkemeier, N. Uygur, U. Pöschl, M. Shiraiwa, M. Kalberer, F. D. Pope, M. K. Kuimova

**Affiliations:** a Department of Chemistry , Imperial College London , London , SW7 2AZ , UK . Email: m.kuimova@imperial.ac.UK; b Department of Chemistry , University of Cambridge , Cambridge , CB2 1EW , UK . Email: markus.kalberer@atm.ch.cam.ac.UK; c Multiphase Chemistry Department , Max Planck Institute for Chemistry , Hahn-Meitner Weg 1 , 55128 , Mainz , Germany; d School of Geography , Earth and Environmental Science , University of Birmingham , Edgbaston , B15 2TT , UK . Email: f.pope@bham.ac.UK

## Abstract

Organic aerosol particles (OA) play major roles in atmospheric chemistry, climate, and public health. Aerosol particle viscosity is highly important since it can determine the ability of chemical species such as oxidants, organics or water to diffuse into the particle bulk. Recent measurements indicate that OA may be present in highly viscous states, however, diffusion rates of small molecules such as water are not limited by these high viscosities. Direct observational evidence of kinetic barriers caused by high viscosity and low diffusivity in aerosol particles were not available until recently; and techniques that are able to dynamically quantify and track viscosity changes during atmospherically relevant processes are still unavailable for atmospheric aerosols. Here we report quantitative, real-time, online observations of microscopic viscosity changes in aerosol particles of atmospherically relevant composition, using fluorescence lifetime imaging (FLIM) of viscosity. We show that microviscosity in ozonated oleic acid droplets and secondary organic aerosol (SOA) particles formed by ozonolysis of myrcene increases substantially with decreasing humidity and atmospheric oxidative aging processes. Furthermore, we found unexpected heterogeneities of microviscosity inside individual aerosol particles. The results of this study enhance our understanding of organic aerosol processes on microscopic scales and may have important implications for the modeling of atmospheric aerosol growth, composition and interactions with trace gases and clouds.

## Abstract 2

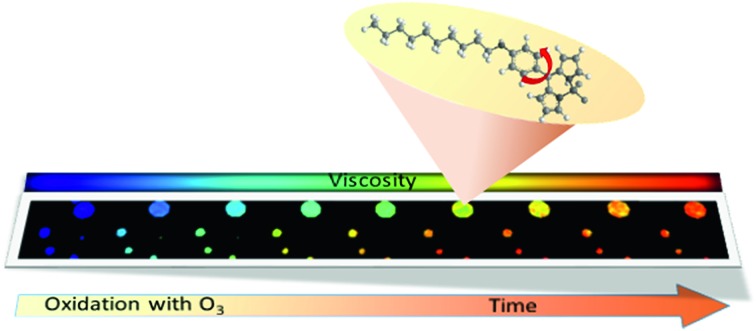
We report quantitative, real-time, online observations of microscopic viscosity changes in aerosol particles of atmospherically relevant composition, using fluorescence lifetime imaging (FLIM) of viscosity.

## Introduction

Organic aerosol particles (OA) play a major role in the atmosphere and climate through absorption and scattering of light, and their interactions with trace gases and clouds.^1^,^2^ Furthermore OA can adversely impact human health since OA are associated with cardiac and respiratory morbidity and mortality.^3^,^4^ Viscosity is an important physical property of aerosol which can determine the ability of chemical species such as oxidants, organics or water to diffuse into the particle bulk thus determining, for example, aerosol composition *via* heterogeneous chemistry.^5^ Most current models assume that lower tropospheric OA are low viscosity liquids, in which the different OA components rapidly partition between the gas and particle phase under thermodynamic control.^6^–^8^ However, recent measurements indicate that OA may be present in highly viscous states^9^–^16^ influencing OA model results.^17^

Several techniques have recently emerged that allow observations of aerosol phase, viscosity and condensed-phase diffusion.^9^,^12^,^14^,^16^,^18^–^20^ In particular, the observation of the particle bounce factor suggested that OA can be present in highly viscous semi-solid states, thereby challenging the traditional views of OA kinetics and thermodynamics.^9^ Likewise, bead tracking and poke flow experiments provided bulk viscosity measurements for α-pinene secondary organic aerosol (SOA) that are broadly consistent with these particles being present in semi-solid states with viscosities in the order of 10^8^ Pa s at 40–50% relative humidity (RH). A more recent study reported diffusion coefficients of water in OA formed through ozonolysis of α-pinene SOA that are several orders of magnitude higher than might be expected from Stokes–Einstein conversion of bulk viscosity measurements,^12^ confirming that small molecules like water do not follow a Stokes–Einstein relation in a viscous matrix of large organic molecules.^21^ However, to our knowledge, direct observational evidence of the microscopic viscosity experienced by individual organic molecules (larger than water) in aerosol particles has not been reported prior to this work. Importantly, no analytical technique that we are aware of can directly quantify and map aerosol viscosity both spatially and temporally in real time. This limitation restricts our ability to observe rapid variations in particle viscosity under atmospherically relevant conditions. Here we quantitatively image dynamic changes in model OA micro-viscosity in real-time, using fluorescence lifetime detection from synthetic molecules, termed molecular rotors.^22^ These fluorescent dyes are sensitive to the micro-viscosity of their environment; with the fluorescence lifetime being directly linked to viscosity (see Methods). Note that microviscosity is the friction experienced by a single particle or molecule undergoing diffusion because of its interaction with its immediate environment. Thus, microviscosity may differ from bulk or macroviscosity that considers resistance of the bulk of the liquid to flow/shear stress. Thus, heterogeneity of the system is another possible cause for the deviation of microviscosity from bulk viscosity.

We recently described the development of FLIM with molecular rotors to μm-sized droplets of inorganic and organic standard compounds showing that the technique can be applied to atmospherically relevant system of low and intermediate viscosity.^23^ Here this methodology is used to investigate the evolution of particle viscosity in two dynamic and atmospherically relevant situations. Firstly, we studied the RH-dependent viscosity of SOA generated from the oxidation of myrcene. Myrcene is a common monoterpene emitted from the biosphere. SOA generated from terpenes represents globally the most abundant type of OAs found in the atmosphere.^24^ One subset of experiments was performed on α-pinene SOA to allow for direct comparison with previous studies.^10^,^12^ Secondly, the effect of atmospheric aging upon aerosol viscosity is exemplified through ozonolysis of oleic acid aerosol. Oleic acid is emitted into the atmosphere in small quantities, through fat burning,^25^ and is often used as a proxy for atmospheric alkenes found in the atmosphere.^26^–^28^

## Results and discussion

SOA was produced through the ozonolysis of myrcene or α-pinene and collected using two widely employed techniques namely a cascade impactor and Teflon filters (Fig. S1† and methods). Terpene and ozone concentrations were significantly above ambient atmosphere levels, which might result in a somewhat different SOA composition compared to ambient SOA. The overall SOA compositional complexity, however, with a wide range of oxidized compounds including carboxylic acids, hydrogen peroxides and carbonyls as well as oligomeric components is similar to ambient conditions as confirmed by mass spectrometry.^29^

Particle collection *via* an impactor allows for direct sample analysis immediately after collection without any further sample preparation assuring that the viscosity is quantified without altering the chemical composition of the airborne particle *via* an extraction step. In fact, the ability to directly image impactor-collected particles is a unique advantage of our approach and was not possible previously. These results were compared to the viscosity data collected on water extracts of SOA particles collected on the Teflon filters, similar to the method employed for example in ref. 10.

**Fig. 1:**
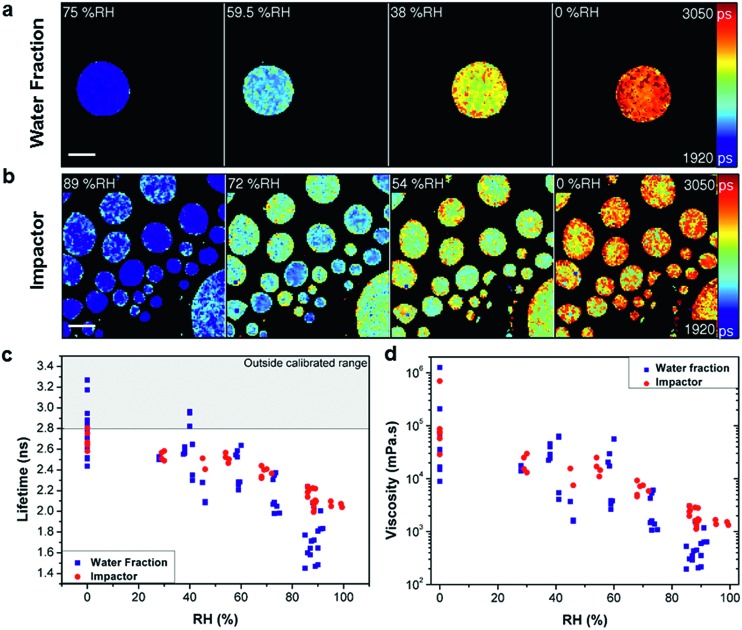
Measuring viscosity of oxidised myrcene aerosol during RH change. Fluorescence lifetime images of (a) water extracted fraction and (b) impactor collected samples at decreasing RH (scale bar 40 μm). Images show the increase of fluorescence lifetime with decreasing RH. (c) Averaged fluorescence lifetimes of individual aerosol particles that were either water extracted (blue) or impactor collected (red), measured at various RHs. (d) Lifetimes converted into viscosity according to the calibration in Fig. S2.† The lifetime values above the threshold in (c) (grey shaded area) are outside the calibrated range for the molecular rotor and were not converted in (d).

The molecular rotor used was Cy3, a hydrophilic dye, which provides a measurable lifetime response to viscosity between 1 and 10^6^ mPa s, Fig. S2.† The SOA fluorescence lifetimes, and hence viscosity, of both the water extracted and impacted particles increase strongly with decreasing RH (89% to 0% RH) as depicted by the colour change in Fig. 1a and b, and shift in lifetime histograms (Fig. S4†). After each change in RH, and prior to measurement, time was allowed for equilibration according to model estimates.^21^

Comparison of the average lifetime (Fig. 1c) and viscosity (Fig. 1d) values for the two collection methods identified an apparent difference in response, most notably at higher RH. We ascribe this to the removal of non-soluble products with lower hygroscopicity in the water extraction sample. Comparison of Fig. 1a and b also identifies that impactor-collected droplets of a similar size show higher intra-particle heterogeneity compared to the water extracted sample, while inter-particle spread is significantly higher in the water extracted sample. The reason for the greater inter particle spread for the water extracted sample is not clear at present, however, we note that preparation of the water extract involves significantly more steps than the impactor sample (collection, extraction, pre-concentration, spraying and viscosity measurement as opposed to just collection and measurement) so there is a greater possibility for analytical variation in the water extract. This data highlights the disparity between the sample preparation methods. Furthermore, Fig. 1c shows that for water extraction samples some lifetime data points measured at or below 40% RH are above our calibration limit of 2.8 ns and these points were not converted to viscosities in Fig. 1d. At the same time, for the impactor-collected data points all recorded values are entirely within our calibration range. We note that the data recorded in the 0–40% RH range likely require longer equilibration times,^21^ than were experimentally feasible. Therefore these data points represent lower limit estimates of viscosity at these conditions. However the particles measured under typical tropospheric RH conditions will be in thermodynamic equilibrium.

The above results clearly indicate that sample preparation without extraction is paramount for a realistic quantification of atmospheric particle viscosity and dynamics, highlighting a unique strength of our imaging approach. The equilibrium average viscosity from both preparation techniques reaches a lower limit viscosity value of 10^4^ to 10^6^ mPa s at 0% RH.

**Fig. 2:**
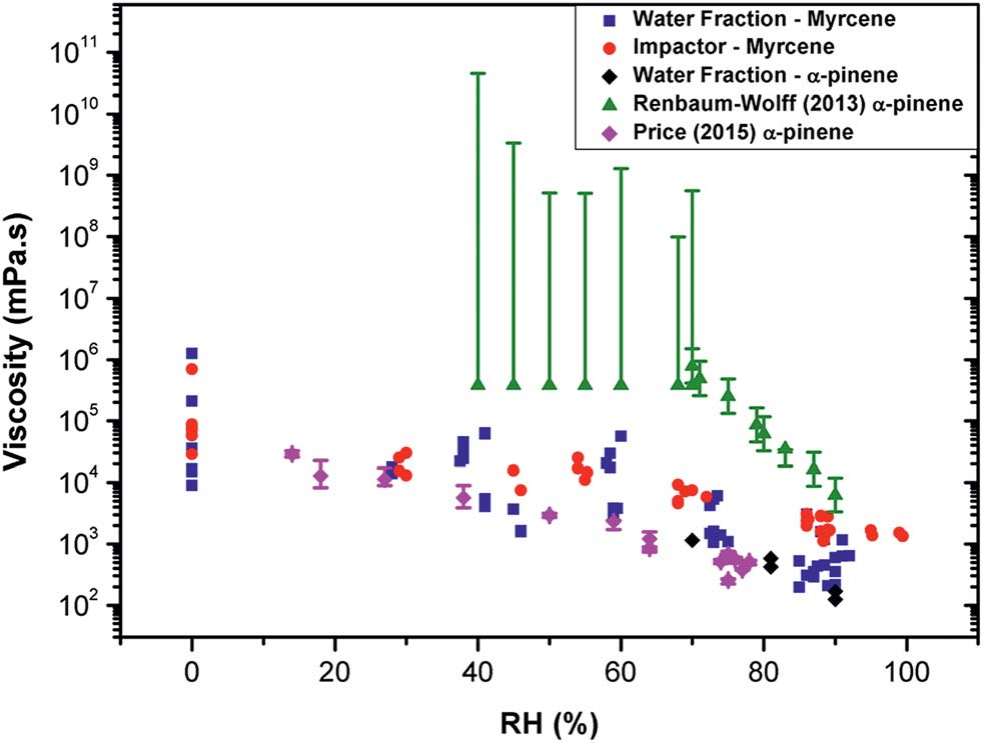
Measuring viscosity of oxidised SOA during RH change using various techniques. The data recorded in this study for myrcene (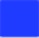 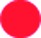) and α-pinene (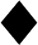), compared to the data in Renbaum-Wolff^10^ and Price^12^ (converted into inferred microviscosity values using the Stokes–Einstein relation) for the water-extracted α-pinene. The viscosities measured in this work of α-pinene-SOA are very close to those for myrcene SOA. While viscosities derived from water diffusion measurements^12^ agree well with the values measured in this work, bulk techniques such as^10^ determine significantly higher viscosities.

We note that our averaged values of viscosity for myrcene at each RH are about 10 times lower than the lowest limit of values reported for water-extracted α-pinene SOA in ref. 10 (Fig. 2). We have measured the lifetime from α-pinene water extracted SOA samples similar to that studied in ref. 10 and confirmed that the lifetime values for α-pinene-SOA are very close to those recorded for myrcene–SOA and thus the nature of the initial chemical substrate (*e.g.* myrcene or α-pinene) plays little role in the discrepancy with the literature.

We hypothesise that the difference can originate from different diffusion scales probed by these techniques. The molecular rotor viscosity imaging approach is sensitive to a very local microenvironment of the probe and will be determined by the domain in which it is localised. It has been previously shown that the diffusion coefficient values calculated from these microviscosities determined using a hydrophobic rotor BODIPY in lipid membranes compare well with those directly determined by fluorescence correlation spectroscopy (FCS), a method that probes diffusion on the microscopic scale.^30^ However, in a highly heterogeneous material it is feasible that molecular rotors do not efficiently incorporate in all domains present in the particle. In particular, it is likely that the Cy3 dye will preferentially incorporate into the more hydrophilic domains. Thus it is likely that the microviscosity values obtained using our method can be different from the bulk viscosity (as in ref. 10 and 13). We note that while the former determines the shape and the bounce efficiency of aerosols, it is likely the latter determines aerosol particle diffusivity and reactivity to chemicals, *e.g.* water transport dynamics within the particle.

This is supported in Fig. 2, which shows the diffusion coefficients of water molecules in water-extracted α-pinene SOA obtained by Price *et al.*^12^ converted into inferred viscosity values using the Stokes–Einstein relation (see ESI† for the details used for conversion) as compared to the bulk viscosity values reported in Renbaum-Wolff *et al.*^10^ and the microviscosity values determined for water-extracted myrcene and α-pinene OA in this work. Even though the Stokes–Einstein relation is not generally applicable for small molecules diffusing through a matrix of large molecules,^19^,^21^,^31^,^32^ the data derived from fluorescence of the hydrophilic molecular rotor overlap well with the viscosity estimated through water diffusivity; and both of the above datasets lie significantly lower than the macroviscosity of α-pinene determined by Renbaum-Wolff *et al.*^10^ Furthermore, diffusion coefficients determined for a hydrophobic molecule pyrene in oxidised α-pinene^16^ coincide well with the data of Renbaum-Wolff, confirming the heterogeneous nature of the oxidised SOA, with very specific hydrophobic and hydrophilic domains that possess vastly different viscosities. We note that our previous studies in viscous lipid membranes using a hydrophobic rotor BODIPY indicated a good correlation between translational diffusion as measured by FSC and rotational diffusion as measured by a molecular rotor.^30^ In combination, these results suggest that the rotational diffusion behaviour of an even larger molecule such as Cy3 in a viscous aerosol matrix is similar to translational diffusion of water. This correlation may be interesting to further explore for fluorophores of different sizes and such studies are currently underway in our laboratory.

**Fig. 3:**
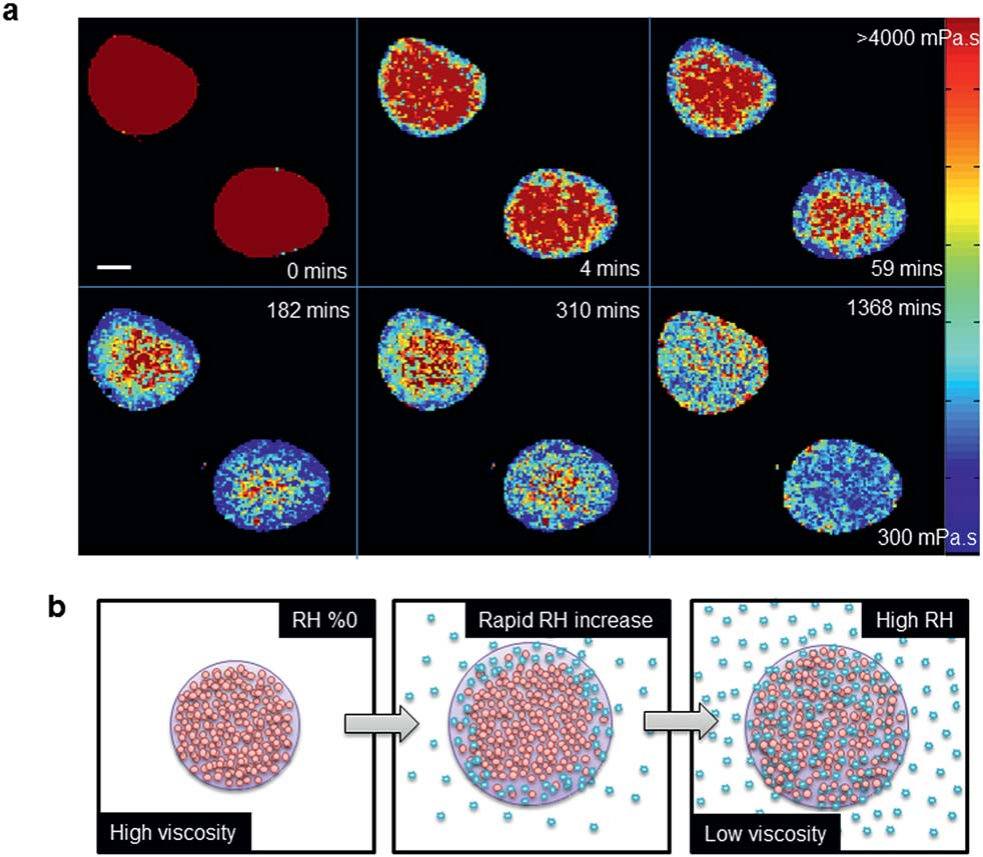
Imaging diffusion fronts of water during hydration of oxidised myrcene aerosol. (a) FLIM images of impactor-collected myrcene droplets obtained between 0–23 h following the RH jump from 0 to 98%, scale bar is 40 μm. (b) A cartoon for the SOA humidification mechanism observed in (a).

The dynamics of the microviscosity change in non-equilibrated impactor-collected myrcene–SOA upon a fast and large jump in RH (0 to 98%) are shown in Fig. 3a. The viscosity reduces more rapidly at the particle surface and a diffusion front forms upon hydration of the high viscosity aerosol as illustrated by Fig. 3b (Movie S1†). This diffusion front is also clearly visible from the changes in width and shape of lifetime histograms (Fig. S5†). Continuous monitoring of the droplets identifies the long equilibration time required for uniformity to be reached (∼23 hours). Theoretical work has previously predicted diffusion fronts occurring in viscous systems.^32^Fig. 3 provides the laboratory proof of this process, by monitoring the dynamics of individual particle hydration in real time. A rough estimate of the diffusion time of the myrcene droplets, shown in Fig. 3, can be obtained using the approach of Shiraiwa *et al.*^17^ which yields an effective diffusion coefficient of 1.5 × 10^–10^ cm^2^ s^–1^ using a characteristic time scale of diffusion of 3 h which is the approximate e-folding time of the penetration of the particle by water. This value is compliant with the recent estimations of Lienhard *et al.* and Price *et al.*, as it lies between those two estimates.^12^,^33^ Such transitional core–shell morphologies, as seen in Fig. 3, might explain the persistent ice nucleation activity of high viscosity organic aerosol particles, even at comparably high temperatures, which has been observed in laboratory studies.^34^,^35^

We note that although water is taken up by the droplets the droplet diameters in our images stay constant (Fig. 3a). We attribute this to the droplet being deposited on a hydrophobic glass surface and thus increased droplet volume due to water uptake results in an increase of the droplet height rather than an increased droplet diameter.

To study the effect of atmospheric oxidative chemical aging upon viscosity, pure oleic acid particles were exposed to a variety of ozone concentrations, from 1.5 to 868 ppm. Whilst oxidized oleic acid particles represent a simple model SOA system, it is noted that they contain the key features governing viscosity in oxidized atmospheric SOA particles: namely, (i) the presence of high molecular weight products due to oligomerization and (ii) compounds with oxidized functional groups such as alcohols, peroxides and carboxylic acids.^29^,^36^–^39^ Thus, oleic acid particles provide a suitable model system to test the effects of atmospheric oxidative processing on particle viscosity with our technique. Furthermore, we tested a large range of ozone concentrations (1.5–868 ppm) to help constrain the kinetic model, which we applied to evaluate our experiments (see below).

BODIPY-C_10_, a more lipophilic dye compared to Cy3, was used as a molecular rotor to measure viscosity during oleic acid oxidation. The calibration of BODIPY-C_10_ was previously reported (Fig. S2†) and it was extensively used for viscosity studies in lipid-rich systems.^22^,^40^,^41^ The BODIPY dye is unreactive to ozone under the conditions of the experiment, see Fig. S7a† and accompanying text.

**Fig. 4:**
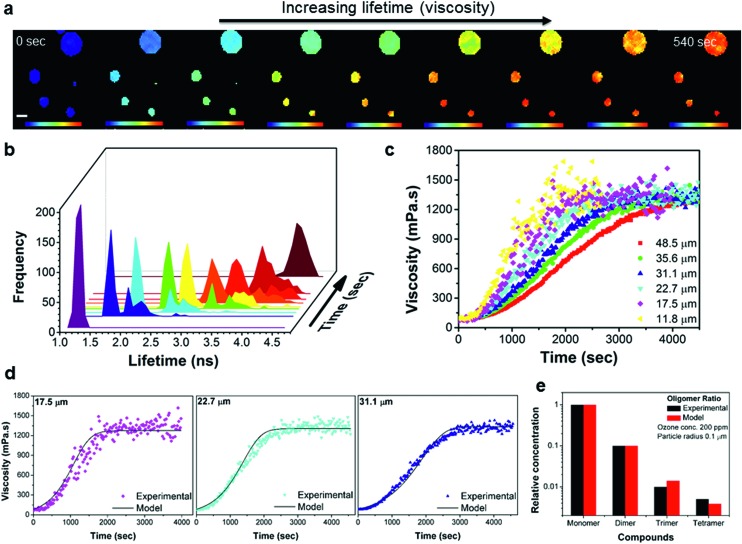
Time-dependent FLIM imaging of oleic acid droplets of varied diameter upon exposure to ozone. (a) FLIM images recorded at 0, 120, 160, 200, 220, 260, 300, 360 and 540 s after the beginning of exposure to 377 ppm of O_3_; rainbow scale under each image represents the lifetime values from 1 to 4.8 ns and scale bar is 40 μm. The high concentration of O_3_ was chosen to optimise the experimental running time, however, the observations remain the same for O_3_ concentrations down to 1.5 ppm. (b) Lifetime histograms extracted from the images in (a). (c) Kinetic response of the individual droplet viscosity to exposure to 12.5 ppm of O_3_ for various droplet sizes. The lifetime recorded in the absence of ozone (Fig. S7†) does not change on this timescale. (d) Selected traces from (c) plotted together with simulated viscosity curves obtained using the KM-SUB model (see ESI for further details†). (e) Experimental monomer:dimer:trimer:tetramer signal intensities normalized to the signal intensity of the sum of all major monomers obtained from a fully oxidised sample using mass spectrometry are shown along with concentration ratios obtained in a corresponding model simulation (see also Fig. S8†).

Spatially resolved FLIM images were acquired in real time before, during and after oxidation. The evolution of particle viscosity through fluorescence lifetime images (Fig. 4a and S6†), and the associated lifetime histograms show an increase in fluorescence lifetime from 1.3 ns to 4.5 ns (Fig. 4b), giving a viscosity increase upon oxidation from *ca.* 80 to >1000 mPa s (Fig. 4c). The kinetics of the ozonolysis is dependent on particle size (Fig. 4c). This is also evident in the FLIM images (Fig. 4a) where the smaller droplets change colour faster than the larger ones. The kinetics of the viscosity change show a size dependence that is proportional to the reciprocal of the radius which suggests surface uptake limitations to ozone uptake as previously reported,^42^,^43^ this is evidenced in Fig. S7e.† All particles approach the same final viscosity, and hence lifetime histogram, indicating that the product composition in completely oxidized particles is similar, irrespective of particle size (Fig. S7e†). However, the final viscosity attained is slightly dependent on the concentration of ozone used (1.5–868 ppm), with lower concentrations leading to somewhat higher viscosities (Fig. S7f†). Higher (and non-atmospherically relevant) ozone concentrations possibly impede secondary chemical reactions thereby reducing the production of high molecular weight (and hence high viscosity) products. These measurements demonstrate that atmospheric chemical aging processes can increase the viscosity of particles, which has the potential to slow down heterogeneous reaction kinetics, and provide a direct first quantification of such the processes.

Given the structures of oleic acid and its oxidation products, we expect the rotor Bodipy-C_10_ to partition effectively in all phases of the final oxidised particle and as such we argue that our measurement of microscopic viscosity is representative of a bulk viscosity value.

Kinetic modelling of the bulk viscosity change during oleic acid oxidation using the kinetic multi-layer model for aerosol surface and bulk chemistry KM-SUB^44^ is shown in Fig. 4d and reproduces the main features of the experimental curves remarkably well. At the same time it largely reproduces the overall compositional distribution of monomeric and oligomeric oxidation products (Fig. 4e) compared to mass spectrometry results. The model provides a set of kinetic parameters, which holds true for a range of ozone concentrations and particle diameters (see Fig. S9 and Table S1†). The modelled oleic acid loss is almost perfectly proportional with the reciprocal of the particle radius. Hence reactive loss scales with surface area which is indicative of a surface or near–surface reaction.

**Fig. 5:**
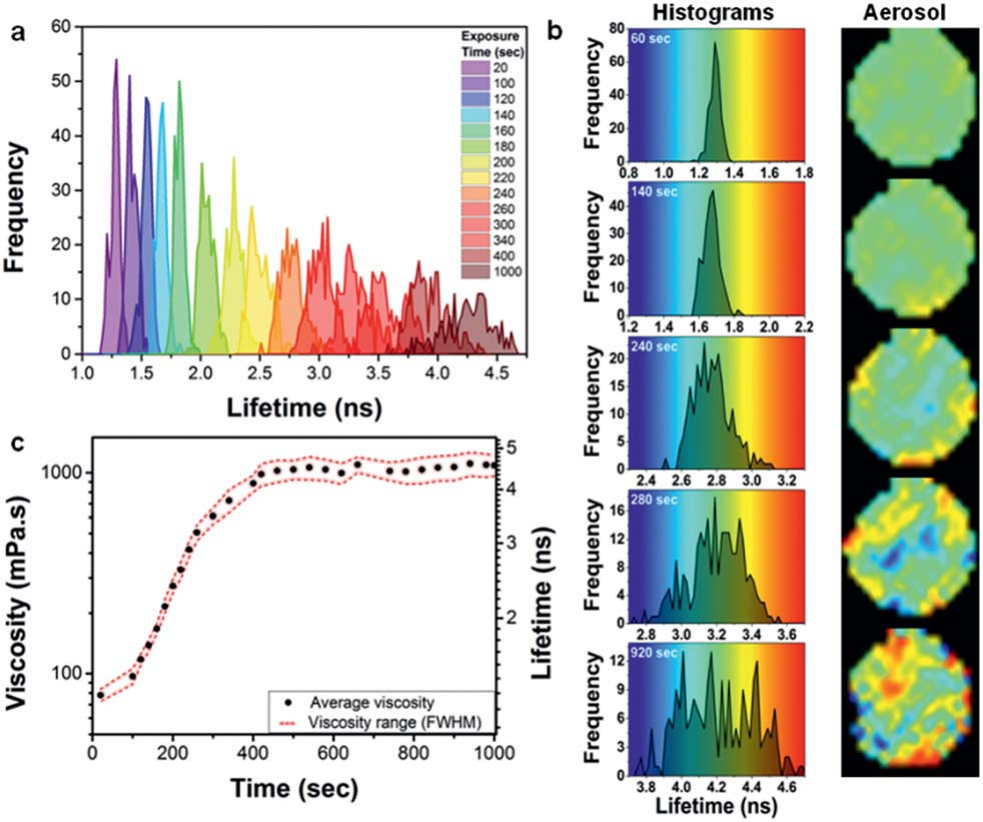
Development of oleic acid droplet heterogeneity with exposure to ozone. (a) Lifetime histograms recorded for an individual droplet (69.4 μm diameter) upon exposure to 377 ppm of ozone for 1000 s. (b) Selected histograms from (a) are shown next to their images, plotted against a rainbow colour hue of 1 ns width (same increment). (c) The increasing average viscosity () and a viscosity spread indicating heterogeneity ( <svg xmlns="http://www.w3.org/2000/svg" version="1.0" width="16.000000pt" height="16.000000pt" viewBox="0 0 16.000000 16.000000" preserveAspectRatio="xMidYMid meet"><metadata> Created by potrace 1.16, written by Peter Selinger 2001-2019 </metadata><g transform="translate(1.000000,15.000000) scale(0.005147,-0.005147)" fill="currentColor" stroke="none"><path d="M0 1440 l0 -80 360 0 360 0 0 80 0 80 -360 0 -360 0 0 -80z M1040 1440 l0 -80 360 0 360 0 0 80 0 80 -360 0 -360 0 0 -80z M2080 1440 l0 -80 320 0 320 0 0 80 0 80 -320 0 -320 0 0 -80z"/></g></svg> ) during ozone exposure; Gaussian fit of histograms in (a) provided peak centres as average viscosity values and the FWHM of histograms provided the spread.

At the final viscosity of ∼1000 mPa s after oxidation, the characteristic diffusional timescale of all components within the particle should still be sufficiently fast to allow for rapid diffusion and a well-mixed system in thermodynamic equilibrium.^21^ However, a striking observation is that the FWHM of the lifetime histograms increases significantly with ozone exposure within individual oleic acid droplets (Fig. 5a), more than could be expected from a natural increase in FWHM with increasing lifetime. This is seen more clearly in Fig. 5b, where the histograms on five stages of exposure from 60 to 920 s are plotted on different scales, but with the same increment corresponding to the same colour hue. The width of the lifetime range is always 1 ns, corresponding to the same colour hue, whereas the actual range changes from 0.8 to 1.8 ns (Fig. 5b – 60 s), 1.2 to 2.2 ns (Fig. 5b – 140 s), *etc.* It can clearly be seen that at the initial stages of oxidation the particle possesses a low degree of heterogeneity (at 60 s exposure FWHM is 7.7% of the peak lifetime), whilst as the oxidation continues a significant heterogeneity appears, evident by the spread of the histogram (at 920 s FWHM is 14.5% of the peak lifetime). An increase in the FWHM is indicative of a greater spread of viscosities and hence more heterogeneity within an individual particle. Since diffusion is non-limiting^21^ then we hypothesise that the observed heterogeneity might be due to liquid–liquid phase separation that has been previously observed for ambient organic particles in the Amazon^45^ as well as laboratory-generated organic/inorganic particles.^46^,^47^ The mass spectrum of fully oxidized oleic acid particles (see Fig. S8†) shows a wide range of species in the mass range *ca.* 100–900 *m*/*z*. Typically the smaller molecules are highly oxidized and hence extremely polar whereas the larger molecules, which are formed by oligomerization, are essentially non-polar.^37^ It seems likely that the small polar molecules will be immiscible with the large non-polar oligomers. The pixel positions of the ‘domains’ of high or low viscosity are non-permanent (Movie S2†). This unexpected observation of high order of intra-aerosol heterogeneity is not explained by diffusion fronts. In future studies, to fully capture the chemistry, and hence the atmospheric ageing of aerosols, this additional level of complexity needs to be accounted for by theory. Given that molecular rotors probe the length scales of the ‘solvent cage’, *i.e.* the volume associated with their rotational diffusion, we expect this heterogeneous viscosity distribution to remain in atmospherically-relevant 100 nm particles, which are too small to be studied with our diffraction resolution-limited technique. At the same time we do not expect these viscous domains to contribute to the ‘bulk’ particle viscosity, manifesting itself in the rate of shape change and/or bounce of the particle and as such their presence is likely to be missed by bulk viscosity measurements.

## Conclusions

The results presented in this study quantify in real-time and on the sub-micrometre size scale changes in the microviscosity of OA model particles of atmospherically relevant composition. Our approach probes microscopic viscosity in the immediate molecular environment of the molecular rotors that depends on their partitioning within the investigated system, which may vary between different types of aerosol particles. Accordingly, we found different relations between microscopic viscosity and bulk viscosity in the two model systems investigated in this work: oleic acid droplets and myrcene SOA. The temporal evolution of microscopic viscosity observed in oleic acid droplets exposed to ozone was consistent with previously reported data and could be reproduced by kinetic model calculations.

In contrast, the microscopic viscosity measured here in myrcene and α-pinene SOA particles was about two orders of magnitude lower than the bulk particle viscosity of SOA particles determined in other studies. However, our experiments still indicate high microscopic viscosity at low RH, which may for example hinder hydroscopic growth and affect ice nucleation pathways.

Using the dynamic nature of FLIM measurements we have detected changes in microscopic viscosity of model aerosol particles due to changes in conditions, such as RH or altered chemical composition due to oxidative ageing. The high spatial resolution of the FLIM technique allowed detecting heterogeneous distributions of viscosity within individual particles, both during hydration of SOA and during oxidation of oleic acid droplets. In the latter case we have detected high viscosity domains maybe indicating unexpected liquid–liquid phase separation.

Overall, our measurement results demonstrate that organic aerosol particles may exhibit a wide range of viscosities, spanning at least four orders of magnitude depending on atmospheric conditions and chemical aging. Our methods and investigations have gone beyond limitations of earlier studies and provide new insights into chemical processes influencing viscosity and into intra-particle heterogeneity and dynamics of microscopic viscosity not previously observed. A more complete understanding of the atmospheric processes controlling aerosol viscosity is needed to assess their effects on heterogeneous chemistry, particle growth and hygroscopicity. An improved understanding of the temporal and spatial evolution of organic aerosol particle viscosity in the atmosphere will allow the incorporation of these processes in atmospheric models to increase our understanding of aerosol processes and effects in the climate system.

## Experimental

### Materials

The molecular rotor *meso*-alkoxyphenyl-4,4′-difluoro-4-bora-3*a*,4*a*-diaza-*s*-indacene (Bodipy-C_10_) was synthesised according to the literature procedure.^40^ The rotor 3,3′-diethylthiacarbocyanine iodide (Cy3, 97%), myrcene, oleic acid and sucrose (all Sigma-Aldrich) were used as received without further purification. Spectroscopic grade solvents glycerol, methanol, dimethyl sulfoxide and chloroform were used. Stock solutions of BODIPY-C_10_ and Cy3 were prepared at a concentration of 5 mM in chloroform and dimethyl sulfoxide, respectively. In experiments where water was required, the water used was doubly distilled.

### Oleic acid aerosol preparation

BODIPY-C_10_ was added from a chloroform stock. The chloroform was then evaporated and the dried dye was dissolved in oleic acid to create the final dye concentration of 10.6 μM. From this mixture, aerosol particles were generated using an airbrush (Model 250-2, Badger) that was pressurised with N_2_ gas. These particles were directed onto hydrophobic siliconized coated coverslips (22 × 22 mm, 0.2 mm thickness, Hampton Research) thereby creating droplets which ranged in size between 5–200 μm on the coverslip. Only droplets with <80 μm diameters were investigated for the viscosity measurements discussed here.

### Myrcene and α-pinene SOA aerosol preparation

Secondary organic aerosol (SOA) particles were produced through the ozonolysis of myrcene or α-pinene vapour within a 1 L reaction vessel (Fig. S1†). Myrcene or α-pinene vapour was generated by flowing N_2_ gas over liquid at room temperature. Ozone was generated by flowing synthetic air through a photolysis tube containing a 254 nm primary output UV light source (Pen-Ray mercury lamp, 90-0004-07) which produces a controllable concentration of ozone (1–1000 ppmv). The ozone concentration (12.5–868 ppm) was varied by obscuring sections of the lamp to reduce the photon flux, and hence production of ozone, and also by modifying the air flow rate through the tube using a mass flow controller (100–500 cm^3^ min^–1^). The ozone concentration was measured using an ozone analyser (49i, Thermo Scientific). The RH was controlled, in the reaction vessel, using a custom-built humidifier. After the reaction vessel, a charcoal denuder was used to remove unreacted ozone from the aerosol flow. Particles were then collected *via* two distinct methods. Firstly, through direct impaction onto glass coverslips using a cascade impactor, and secondly, onto a Teflon filter. A differential mobility analyser and a condensation particle counter (TSI, 3081, 3775) were used to measure the particle size distribution. The gas flow rates of N_2_ and air were optimised to ensure a high concentration of particles with large diameters. Gas flow rates for N_2_ and air of 187 and 180 ml min^–1^, respectively, produced a total aerosol concentration of SOA of about 180 mg m^–3^ with a modal diameter of about 200 nm.

### Impactor SOA sampling

The SOA particles generated as described above were collected on glass coverslips (*Ø* 12 mm, 0.12 mm thick, Hampton Research) within a cascade impactor, which has previously been described in detail elsewhere.^48^ Briefly, the 12-stage impactor was operated at a flow rate of 8.5 L min^–1^ (*i.e.* 0.37 L min^–1^ sample flow and 8.13 L min^–1^ clean air make up flow) and the particles collected on stages 4 and 5 were used for the FLIM analysis. Prior to the collection of the SOA aerosols, the Cy3 molecular rotor was deposited in a thin film on the coverslips by repeatedly dipping the slides (4–6 times) in a solution of Cy3 in chloroform (25 μM solution). The chloroform solvent was subsequently removed by evaporation. The RH in the flow set-up was controlled by the custom-built humidifier and was held constant at ∼50%. After a typical collection time of 8 min, the cover slips were removed from the impactor, stored in an air-tight box at room temperature and the lifetimes were measured by FLIM within 10 hours of collection. The z-stack measurements confirmed that the fluorescence intensity is homogeneous in ‘slices’ obtained of aerosol particles up to 50 μm up from the coverslip, hence Cy3 is uniformly distributed in the droplet bulk. This method results in a direct collection and analysis of the entire SOA particles. We note that such samples might be prone to evaporation of semi-volatile components between collection and analysis.

### Water extract SOA sampling

Water extracted SOA, again produced using the method described above, were collected on a Teflon filter (MITEX PTFE 5.0 μm, WH PL 47 mm). The RH was set to <2% by removing the humidifier from the set-up. The samples were collected for 6 hours, after which the filters were stored at –20 °C for less than 72 h before FLIM experiments were performed. Prior to FLIM analysis, filters were cut into small pieces, placed in 7 ml of high purity water (ROM II Ultra-Pure Solvent) and sonicated for 15 min. The filter fragments were removed and the water extract was stored at 2 °C until further use (<14 h). In this method, Cy3 was added to the aqueous SOA extract immediately prior to imaging, to create a dye concentration of *ca.* 15 μM. The solution was then sprayed onto glass coverslips (*Ø* 12 mm, 0.22 mm thick, Hampton Research) using a disposable plastic pump spray bottle. This method relies on the organic aerosol components being fully extracted by sonication in water and therefore represents a more indirect method of SOA analysis, as potentially not all SOA mass is extracted from the filter.

### Calibration of viscosity probes

For each aerosol system (oleic acid and myrcene–SOA), a suitable molecular rotor was selected based on hydrophobic/hydrophilic match of the aerosol to molecular rotor. For the hydrophobic oleic acid aerosol the most suitable probe was BODIPY-C_10_, which has previously been successfully used for lipid based systems, *e.g.* membranes of live cells,^40^,^49^ model lipid bilayers^50^ and encapsulated lipid microbubbles.^41^ The fluorescence intensity and lifetime of BODIPY-C_10_ was demonstrated to be sensitive across a wide range of viscosities 1 to 10^4^ mPa s.^50^ We have previously demonstrated that fluorescence lifetime is a superior marker for microviscosity, compared to fluorescence intensity, since it is not sensitive to gradients in the probe concentration.^22^,^49^

The fluorescence lifetime decays of BODIPY-C_10_ and Cy3 were measured in mixtures of methanol/glycerol and sucrose/water, at a working dye concentration of *ca.* ∼10 μM. The dye mixtures were measured in quartz cuvettes (BODIPY-C_10_) and 8-well μ-Slide chamber (Ibidi) (Cy3) *via* time correlated single photon counting (TCSPC). BODIPY-C_10_ decays were measured on a Jobin Yvon IBH data station (5000F, HORBIA Scientific Ltd.) using a 467 nm 1 MHz pulsed NanoLED (N-467, HORBIA Scientific Ltd.) for excitation. Emission was captured at 515 ± 5 nm with a long pass filter at 470 nm until a peak count >10 000 was reached; 1024 ADC and collection rate <2% was maintained. Cy3 decays were measured using the FLIM system (see below). The calibration plot for BODIPY-C_10_ is linear between *ca.* 5 and 1500 mPa s (ref. 50) and follows the Förster Hoffmann equation for fluorescence lifetime^22^,^51^ (eqn (1)).
1

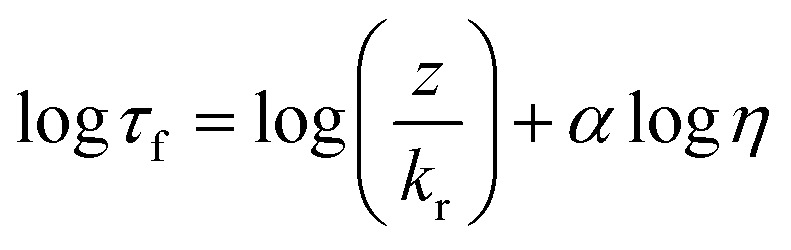

where, *τ*_f_ is the fluorescence lifetime, *k*_r_ is the radiative rate constant, *η* is the viscosity and *z* and *α* are constants. For BODIPY-C_10_ in methanol/glycerol mixtures in the viscosity range 5–1500 mPa s (Fig. S2a†) this equation becomes:

2 log *τ*_f_ = 0.4569 log *η* – 0.75614

where, *τ*_f_ is the fluorescence lifetime of the molecular rotor in ns and *η* is viscosity in mPa s. The BODIPY-C_10_ decays, at all viscosities, are monoexponential as shown in Fig. S2c.† For the myrcene SOA aerosols the Cy3 dye was chosen as the molecular rotor, due to its good aqueous solubility. Cy3 was previously successfully used to determine microviscosity in the cell cytoplasm^52^ and in model sucrose aerosols.^23^

Previously we have shown that Cy3 lifetimes do not follow the Förster Hoffmann eqn (1) at viscosities greater than 30 mPa s.^23^ For this work, the Cy3 viscosity–fluorescence lifetime response was calibrated for the viscosity range 1 to 10^6^ mPa s (Fig. S2b†) using calibrant solutions of sucrose in water at concentrations up to 80% w/w sucrose. The time resolved fluorescence signal from Cy3 is found to be biexponential over all viscosities probed (Fig. S2d†). The mean fluorescence lifetime can be linked to viscosity through the use of a Hill function^53^ shown in eqn (3). Where the mean fluorescence lifetime, *τ*_f_, is the intensity-weighted mean, defined as (*A*_1_*τ*_1_^2^ + *A*_2_*τ*_2_^2^)/(*A*_1_*τ*_1_ + *A*_2_*τ*_2_), where *τ*_1_ and *τ*_2_ are individual fitted exponential lifetimes, and *A*_1_ and *A*_2_ are their relative amplitudes as percentages. The relationship between the mean fluorescence lifetime data for Cy3 and viscosity is accurately described by the Hill function, eqn (3), Fig. S2b.†

3 *τ*_f_ = 2.8054*η*^0.475^(11.436 + *η*^0.475^)^–1^

The viscosity of methanol/glycerol mixtures for the entire calibration range was measured with a viscometer (Stabinger Viscometer SVM 3000, Anton Paar). The Cy3 calibration solutions, that used non-saturated sucrose concentrations (<67% w/w), were prepared by mixing increasing concentrations of sucrose in water and adding <0.5% Cy3 stock, and the viscosities were measured using a rheometer (HR03, TA Instruments). However, to achieve higher viscosities, supersaturated sucrose/water solutions were prepared, with sucrose concentrations >67% w/w; for these the direct rheological measurements were impossible due to sucrose precipitation. Instead, the theoretical model using Gènotelle's equation^54^ was used to predict the solution viscosity. The supersaturated solutions were prepared by controlled water evaporation, in which solutions of 40% (w/w) sucrose/water (*ca.* 5 ml) containing <0.2% Cy3 stock were heated at 100 °C in a round bottom flask under vacuum (150 mbar) for between 5–10 min. The water activity in each sample was determined using a Karl Fischer titrator (Mettler Toledo).^55^ The known water percentage in the sample allowed Gènotelle's equation to be applied, as shown in eqn (4).log_10_ *η*/η* = *a*_1_ + *a*_2_*x* + *Φ*(*b*_1_ + *b*_2_*x*^*n*^);

4 *Φ* = (30 – *t*)/(91 + *t*)

where, *η* is the dynamic viscosity, *x* is the mole fraction of sucrose, η* is standard viscosity (1 mPa s), *t* is temperature (°C), *Φ* is calculated from the known temperature as shown above and *a*_1_, *a*_2_, *b*_1_, *b*_2_, *n* are constants, *a*_1_ = –0.1245, *a*_2_ = 22.452, *b*_1_ = 1.095, *b*_2_ = 46.39 and *n* = 1.303.^54^

### Atmospherically controlled environmental chamber

We have previously reported using molecular rotors for imaging of model aerosols in static FLIM experiments, where aerosols were produced off-line and subsequently measured on the microscope.^23^ Here we have coupled the aerosol production with online FLIM detection, taking advantage of unique capability of our methodology to image viscosity dynamically, as the changes occur. The spatially resolved measurements of fluorescence lifetimes of molecular rotors in aerosol droplets were performed in our custom designed environmental aerosol chamber which was mounted onto the microscope stage, Fig. S3.† Oleic acid or myrcene SOA droplets were deposited onto glass coverslips (dimensions: 50 × 25 × 20 mm) which were mounted on the bottom of the chamber (Fig. S3†) using high vacuum grease (Dow corning) for adhesion. The chamber allowed for the control and variation of both ozone concentration and RH during the online FLIM experiments. The desired RH in the chamber was generated by mixing dry and humidified air using two mass flow controllers (MKS, 0–500 sccm). RH (0–90%) was monitored *via* a digital humidity sensor (SHT075, Sensirion) located within the chamber. Equilibration times of droplets were based on the estimates from the model calculations,^21^ which accounts for both particle size and viscosity. The equilibration times were <30 min at RH > 90%, 20–100 min at intermediate RH of 40–90% and >100 min for RH < 40%. The equilibration time for myrcene SOA water extracts was assumed to be sufficient once the particle shape no longer changed; this was based on our observation that at the lower RH droplets reduced in diameter. Ozone was produced and monitored, as described above, and was delivered to the chamber's inlet exposing the oleic acid droplets to ozone (Fig. S3†). At the chamber exit, ozone was removed by passing the gas through a 1.5 m long charcoal denuder.

### FLIM viscosity measurements of aerosols

 Eqn (2) and (3) provide the direct means for converting the fluorescence lifetime of the molecular rotor to the environmental microviscosity within aerosol particles, in both a spatially (100 s nm) and a temporally (subminute) resolved manner. The atmospheric chamber was mounted onto the microscope stage of an inverted scanning confocal microscope (SP5, Leica Microsystems Ltd.) and an x63 (N.A. 1.2) HCX PL APO CS water immersion objective lens with correction collar (11506279, Leica Microsystems Ltd.) was used for imaging. Brightfield and intensity localisation imaging was achieved by excitation at 488 nm (Bodipy-C_10_) and 561 nm (Cy3), whilst emission was captured between 500–660 nm (Bodipy-C_10_) and 570–700 nm (Cy3). Droplet size was measured using LAS software (Leica Microsystems) by drawing line plots across the centre of the aerosol and the edge was determined from the change in brightfield intensity.

Lifetime images were acquired using a time correlated single-photon counting (TCSPC) module (SP830, Becker & Hickl GmbH) and tuneable multi-photon laser as an excitation source (680–1080 nm, 80 MHz, 140 fs, Vision II, Coherent Inc.). Two-photon excitation of aerosols was performed at 840 nm and laser power maintained <200 mW prior to entering the microscope with an image format of 128 × 128. Cy3 lifetime images were acquired until a peak count of 500/pixel was reached across the majority of the image. It was established that the lifetime of Bodipy-C_10_ in oleic acid could change upon prolonged laser exposure, possibly due to the oxidation of the oleic acid, and so the following low power settings were used in these experiments: <100 mW, 64 × 64 image size, and collection times of 20–30 s. This ensured that the Bodipy-C_10_ lifetime in oleic acid in an air environment did not change over a period of up to 24 hours. Lifetime images were analysed using either the TRI2 software (for Bodipy-C_10_) (Version 2.7.6.1, Gray Institute for Radiation Oncology and Biology)^56^ or FLIMfit (for Cy3, where offset correction was necessary, see below) (OMERO) and decays fitted using monoexponential (Bodipy-C_10_) or biexponential (Cy3) models. We have checked for multiple samples that TRI2 and FLIMfit produces identical results for samples, where the offset correction was not an issue.

### Lifetime analysis of aerosols

The instrument response function (IRF) was recorded using the second harmonic generation signal from a dried urea sample. Bodipy-C_10_ FLIM data was exported and analysed in TRI2 software. A mono-exponential model was fitted to each image pixel using the Levenberg–Marquardt algorithm and lifetime images were produced. The oleic acid kinetic response was analysed by individually masking a single droplet within the image and binning all the pixels to derive an average lifetime of that droplet at that time point. A batch fitting function was used to perform this task for all time points^57^ and then this method was applied to each droplet. Due to a short acquisition time a minority of data had low peak counts so only data with >30 peak counts and a reduced chi-squares 0.8 > *χ*_r_^2^ < 1.2 were accepted as final values; thresholding was used to remove background noise prior to analysis. A false rainbow colour scale was assigned to each fluorescence lifetime value in FLIM images: blue for a short lifetime and red for a long lifetime, to provide lifetime maps.

Cy3 FLIM data (images and individual calibration decays) were exported and analysed in FLIMfit software (OMERO). Due to the long decay lifetimes, decays were incomplete within the acquisition time window, which did not allow the direct fitting of an offset parameter for each trace accurately. To remove the offset (and hence the uncertainty in the lifetime values), the dark counts were subtracted from both the IRF and the individual decay traces by recording a background signal without the sample on the microscope stage. A Matlab code was written to remove the background from each image by subtracting the corresponding dark counts from any pixel that had counts above zero. The data was then analysed using the biexponential decay model and Levenberg–Marquardt algorithm without an offset fitting parameter.

## Supplementary Material

Supplementary information

Supplementary movie

Supplementary movie
