# Supplementary material for: Direct imaging of changes in aerosol particle viscosity upon hydration and chemical aging
Source: Chem Sci. 2015 Nov 12;7(2):1357–67. doi: 10.1039/c5sc02959g (PMC5975791; doi:10.1039/c5sc02959g)
Supplement: Supplementary file 1 [file SC-007-C5SC02959G-s001.pdf]

Electronic Supplementary Material (ESI) for Chemical Science.
This journal is © The Royal Society of Chemistry 2015

Supporting Information

Direct on-line imaging of changes in aerosol viscosity upon atmospheric hydration and chemical aging

Neveen A. Hosny[a], Clare Fitzgerald[b], Aurimas Vyšniauskas[a], A. Athanasiadis[a], Thomas Berkemeier[c], Nihan Uygur[c], Ulrich Pöschl[c], Manabu Shiraiwa[c], Markus Kalberer[b]*, Francis D. Pope[d]* and Marina K. Kuimova[a]*.

[a] Department of Chemistry, Imperial College London, London, SW7 2AZ, UK. [b] Department of Chemistry, University of Cambridge, Cambridge, CB2 1EW, UK. [c] Multiphase Chemistry Department, Max Planck Institute for Chemistry, Hahn-Meitner Weg 1, 55128 Mainz, Germany. [d] School of Geography, Earth and Environmental Science, University of Birmingham, Edgbaston, B15 2TT, UK.

SI Materials and Methods

Individual droplet heterogeneity analysis. The influence of ozone oxidation on oleic acid droplet heterogeneity was examined at an ozone concentration of 377 ppm (Figure 4). Individual droplet lifetime histograms were extracted by separately masking each droplet and producing lifetime images. Lifetime histograms were exported to Origin and peak fitting analysis performed by applying a single Gaussian peak to each histogram where the full width half maximum (FWHM) was used to represent the spread of lifetimes.

Mass spectrometry analysis of products generated from oleic acid ozonolysis. The reaction products formed in the aerosol formed by the ozonolysis of oleic acid were characterised using an ultra-high resolution mass spectrometer (LTQ Orbitrap Velos mass spectrometer, Thermo Scientific) in direct infusion negative ionisation mode, with a heated electrospray ionisation source (Thermo Scientific). Using the same particle collection method as for the water-extracted samples the filter was extracted into 0.8 ml water (LC/MS grade) and 0.8 ml acetonitrile. The solution was stored at 4°C prior to mass spectrometry analysis (<14 h). The optimal electrospray settings for the mass spectrometer were: heater temperature = 100°C, sheath gas flow = 15 a.u., spray voltage = 3.4 kV, capillary temperature = 275°C, injection rate = 7 μl/min; no sweep gas was used. The API stack, ion optics and Orbitrap mass analyser were set to the manufacturer's default settings.

Kinetic Modelling of aerosol surface and bulk chemistry. The kinetic multi-layer model for aerosol surface and bulk chemistry (KM-SUB) [1] has been used to model the temporal evolution of viscosity during oleic acid ozonolysis. KM-SUB treats the following processes explicitly: gas-phase diffusion, adsorption and desorption, surface-bulk exchange, bulk diffusion, and chemical reactions at the surface and in the bulk. In this study, the model adopts the following chemical mechanism of oleic acid ozonolysis [2,3].

(R1)	O_3 + oleic acid → c*(NN + CI) + (1-c)*(C_9 + CI)	$k_{BR,1}$
(R2)	CI → C9	$k_{BR,2}$
(R3)	CI + CI → CI dimer	$k_{BR,3}$
(R4)	CI + C9 → dimer	$k_{BR,4}$
(R5)	CI + dimer → trimer	$k_{BR,5}$
(R6)	CI + trimer → tetramer	$k_{BR,5}$
(R7)	CI + tetramer → pentamer	$k_{BR,5}$

In R1, oleic acid reacts with ozone (O_3) to form either nonanal (NN) or another not further distinguished C_9 compound (C9, a lumped surrogate of azelaic acid, nonanoic acid, 9-oxo-nonanoic acid) and a Criegee intermediate (CI). The branching ratio between NN and C9 is described by the stoichiometric coefficient c. The highly volatile nonanal is assumed to evaporate instantaneously once it reaches the particle surface. The reactive CI is lost by first-order decay to C9 (R2) or by recombination with another CI (R3). The CI initialises the oligomerisation reaction by formation of a reactive dimer (R4) that undergoes further oligomerisation with CI (R5-R7). Oligomerisation is treated as occurring with a uniform oligomerisation rate constant $k_{BR,4}$ until the pentamer level. Higher oligomers have been neglected since these are assumed to form in insignificant amounts [4]. The viscosity of the organic mixture v_{mix} has been calculated using an Arrhenius approach, Equation 5 [5].

$$v_{mix} = \exp(\sum x_i \log(v_i)) \quad \textbf{[5]}$$

where x_i and v_i are the pure components' mass fraction and viscosity, respectively. The viscosity of oleic acid is 0.083 Pa.s [6], which can be converted to a bulk diffusion coefficient using the Stokes-

Einstein equation. The viscosity and the bulk diffusion coefficient of monomeric compounds (NN, CI, C9) are assumed to be the same as of oleic acid and have been fixed to these values during the optimisation. The viscosity of the pure oligomer compounds has been approximated to scale in a power law relationship with chain length. The scaling factor f_{visc} was used as a fitting parameter since no experimental data is available for the products in this study ($v_{trimer} = v_{dimer}\ (M_{trimer}/M_{dimer})$^$f_{visc}$, $v_{tetramer} = v_{dimer}\ (M_{tetramer}/M_{dimer})$^$f_{visc}$, etc.).

O_3 reacts with oleic acid while diffusing into the particle, creating a steep gradient from particle surface to bulk. This scenario is often referred to as the classical reacto-diffusive case e.g. Hanson et al. 1994 [7]. The sub-surface bulk layer resolution of the kinetic model is adjusted to be 1/3 of the reacto-diffusive length, which is the distance an ozone molecule travels on average before reacting with an oleic acid molecule. In total, particles have been described with 30 layers in this study.

Bulk diffusion of oleic acid occurs sufficiently fast in the liquid aerosol particles so that no concentration gradient is observed [1]. Bulk diffusion coefficients of compounds below the dimer level have thus been lumped to a value of $D_{b,monomer} = 1.00\times10^{-7}$ $cm^2\ s^{-1}$ that ensures perfect mixing. The diffusion coefficient of the oligomeric species, $D_{b,oligomer}$, has been used as a fitting parameter to preserve the possibility of diffusive limitation to the reaction rate. Surface reaction rates k_{SLR} are assumed to scale linearly with a factor of f_{SLR} to their respective bulk reaction rate constants k_{BR} ($k_{SLR} = f_{SLR}\ k_{BR}$).

The numerical values of the kinetic parameters required for KM-SUB are summarised in Fig. S9. They have been obtained by a global optimization method that combines a uniformly-sampled Monte-Carlo search with a Genetic Algorithm (MCGA). This MCGA method first samples a large number of randomly generated kinetic parameter sets and evaluates their (least squares) deviation to multiple experimental data sets. Data sets with highest correlation are picked and then further optimized using a genetic algorithm. The experimental data sets to which the model has been optimised were viscosity data (this study), degradation of oleic acid concentration data [8] and oligomer ratios [4]. These data sets cover a wide range of particle size and ozone concentration, a necessary condition to restrain the kinetic regime of the reaction system [9]. It can be seen that KM-SUB reproduces all the experimental results very well with a single set of kinetic parameters, as shown in Table S1.

The oleic acid – ozone reaction mechanism has been investigated extensively in the past (e.g. ref [2,3]), but too complex mechanisms would leave to much freedom to the kinetic model. The model mechanism is hence kept as simple yet compliant with the current literature as possible (Eqs. R1-R7). As a result, the kinetic parameters of the model have to be seen as effective parameters of simplified processes and their numerical value should not be over interpreted. The range of the kinetic input parameters however was constantly monitored and cross-checked with literature estimates if available and we find no unrealistic kinetic parameters. Rate constants were determined to be in the range of 1×10^{-18} to 2×10^{-16} cm^3 s^{-1}; diffusion coefficients did not fall below 1×10^{-9} cm^2 s^{-1}, still corresponding to a liquid solution. Henry's law coefficient lies within a factor of 1.5 with what has been estimated before in the literature [10].

During the model building process, the chemical mechanism had to be iterated several times until a good agreement with the experimental data could be obtained. The initial reaction of oleic acid and ozone is well-described by a near-surface bulk reaction producing Criegee intermediates. The model reproduces oleic acid decay data fully (ref [8], Fig. S9). Criegee intermediates play a central role as they function as building blocks for the oligomers, which are responsible for the viscosity increase. This general idea is compliant with the observed product distribution showing a gradual decrease in the abundance of oligomers with increasing length (Fig. 4e). The model fitting determines the relative rates of CI oligomerization vs. CI removal that leads to such a distribution. The fact that higher order oligomers affect the viscosity much stronger than lower order oligomers, a result returned from the model fitting, explains the time delay between oleic acid consumption and increase in viscosity that leads to the s-shape in the measured viscosity data versus time. A mechanism where reactive intermediates are added subsequently to existing oligomers is further backed up by the fact that the final viscosity is mostly independent of particle size and oxidant concentration: this can only be understood when the processes leading to oligomerization are all pseudo-first order i.e. one reactant (here: oligomers) being available in excess over another very reactive compound (here: Criegee intermediate).

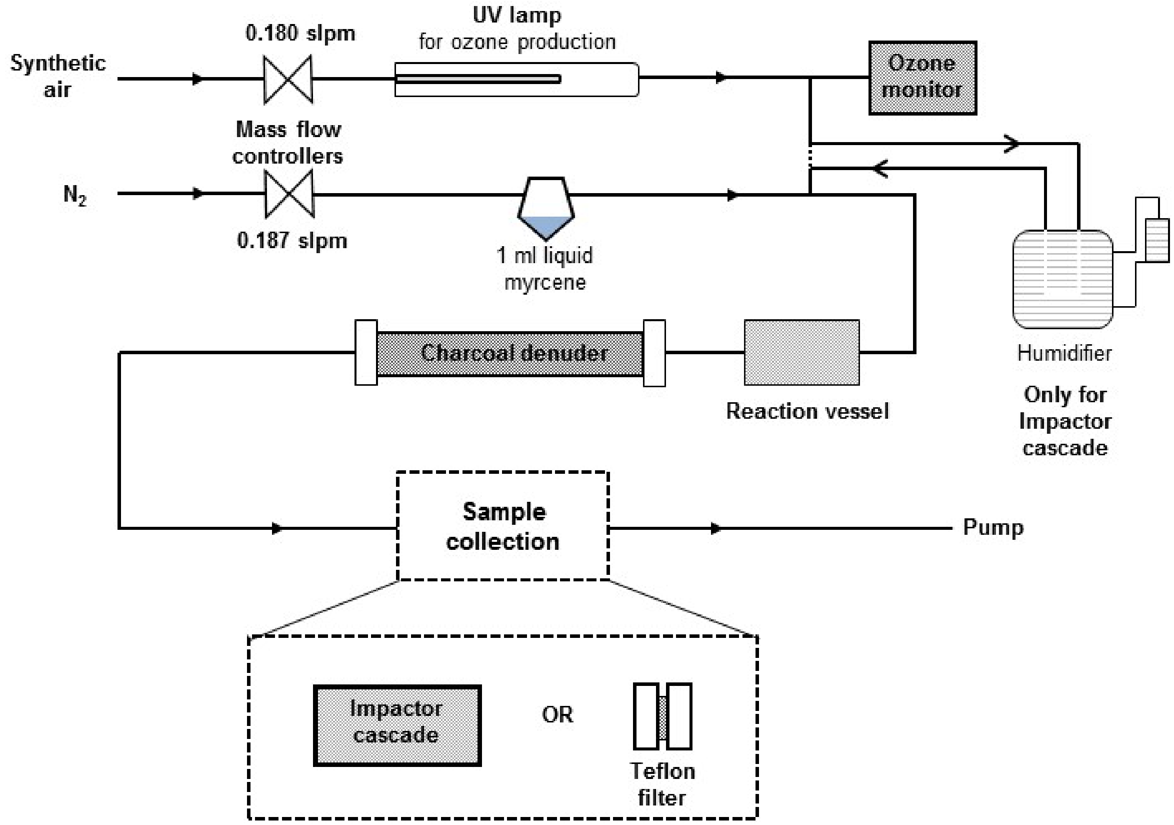

Fig. S1: Schematic diagram of the myrcene SOA production. Two collection methods were used in this work: a cascade impactor or a Teflon filter.

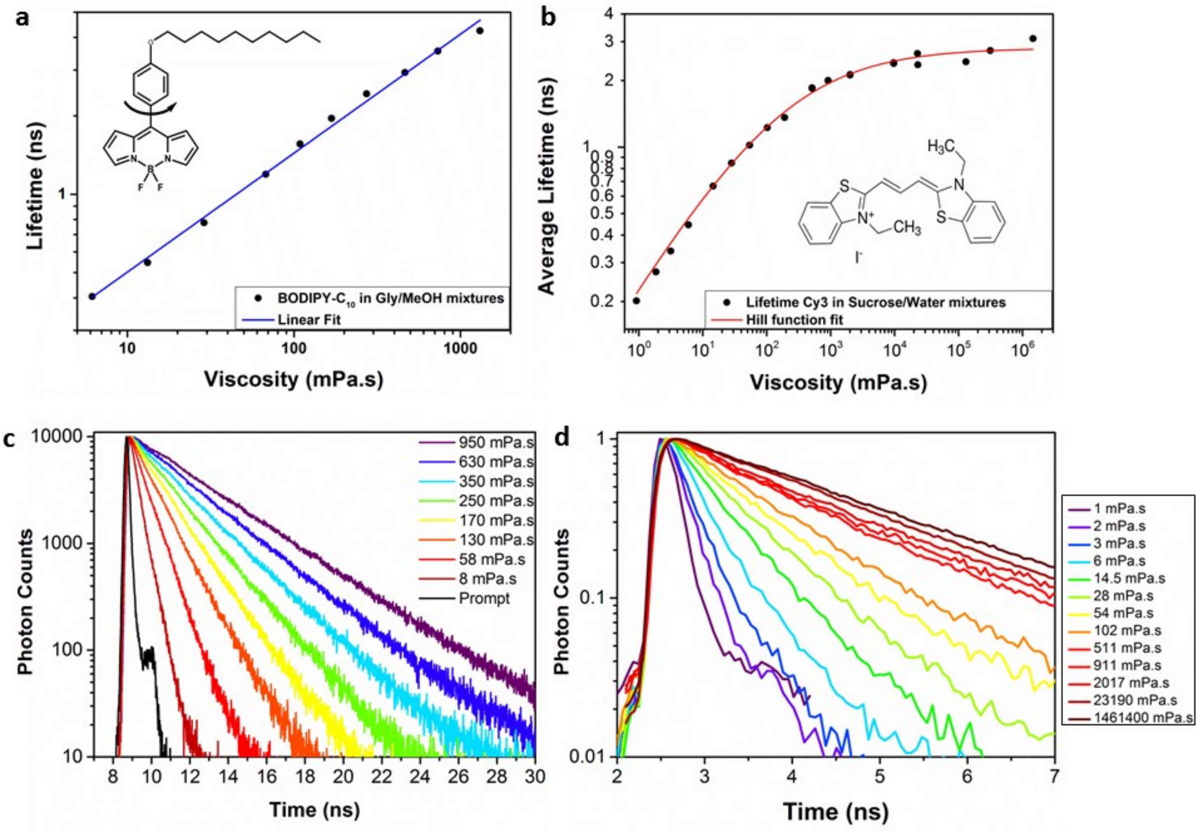

Fig. S2: Calibration of the molecular rotor lifetimes in solutions of increasing viscosity with chemical structures. a) BODIPY-C_{10} in methanol/glycerol mixtures [11]. b) Cy3 in sucrose/water. Lifetime decays of BODIPY-C_{10} (c) and Cy3(d) in these solutions are also shown.

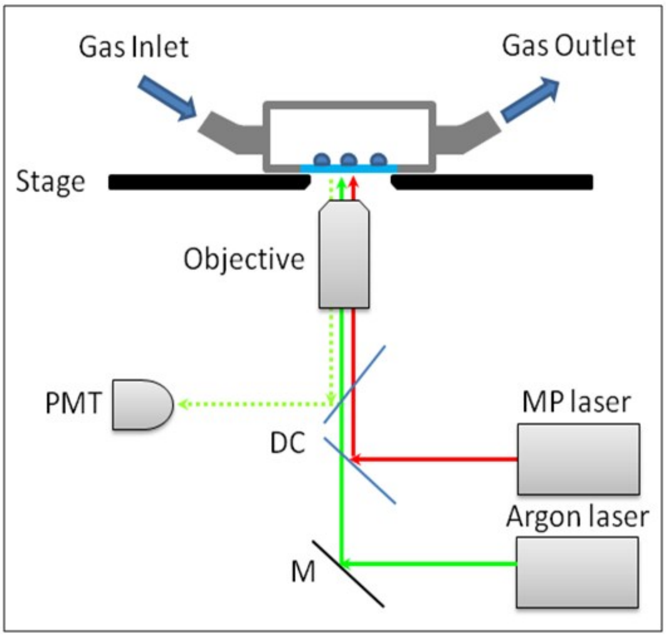

Fig. S3: Schematic diagram of the environmental imaging chamber and simplified optical set up. The inlet and outlet for gas delivery to the chamber allow for the RH and ozone concentration control. An excitation of the aerosol droplets placed on a glass coverslip on the bottom of the chamber is possible using either a pulsed multi-photon (MP) laser or a CW argon laser. The resulting emission is reflected to a photomultiplier tube (PMT).

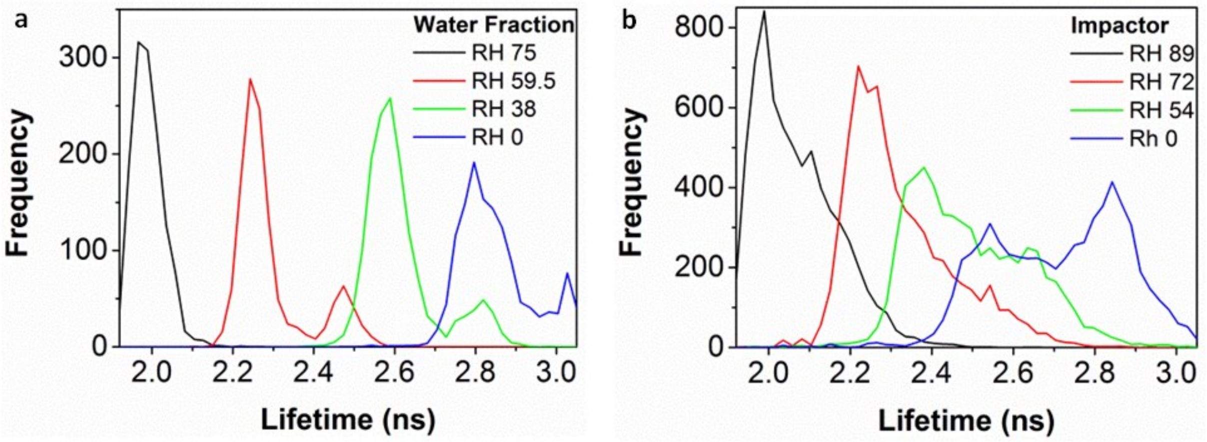

Fig. S4: Fluorescence lifetime histograms of oxidised myrcene SOA particles exposed to decreasing relative humidity (RH, %) show a significant increase in the histogram width upon RH change from 89% (impactor) or 75% (water fraction) to 0% RH. This indicates a significant increase in the heterogeneity of the more viscous aerosol samples at lower RH. Comparison of the two collection methods water extraction (a) and impactor (b), show a broader spread in lifetime recorded from (b), which indicates a more heterogeneous system obtained in this collection method. The histograms correspond to images in Figure 1.

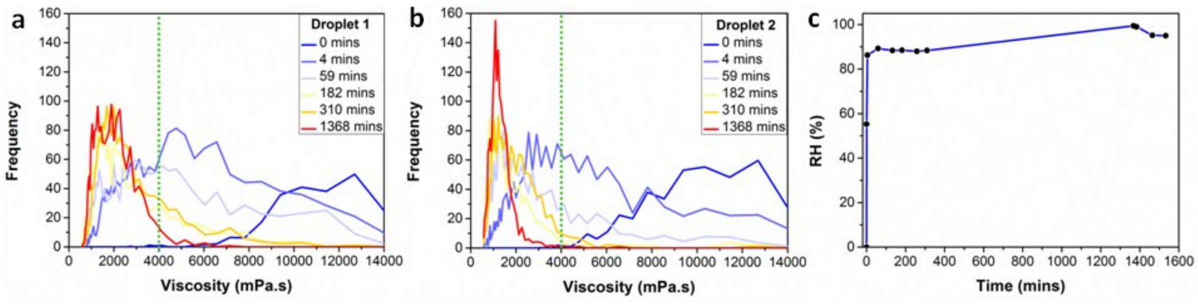

Fig. S5: The response of lifetime histograms of two myrcene SOA droplets in the impactor samples to the RH jump from 0 to 98%. Green dotted line signifies cut-off for the colour hue in Figure 2, main

text. a) Droplet 1 (upper left corner of images in Figure 2), b) Droplet 2 (lower right corner of images in Figure 2), c) Temporal profile of RH change during this experiment. The histograms correspond to images in Figure 2a. The histograms cannot be described by a single Gaussian peak and hence they indicate the presence of at least two distinct viscosity domains within each SOA droplet. This is a signature of a non-equilibrium state in each droplet and the formation of the diffusion front, which disappears only after 23h of exposure. The effect of RH on the heterogeneity observed in myrcene aerosols is also visualised using a video (Supplementary Video 1) over 0-23h.

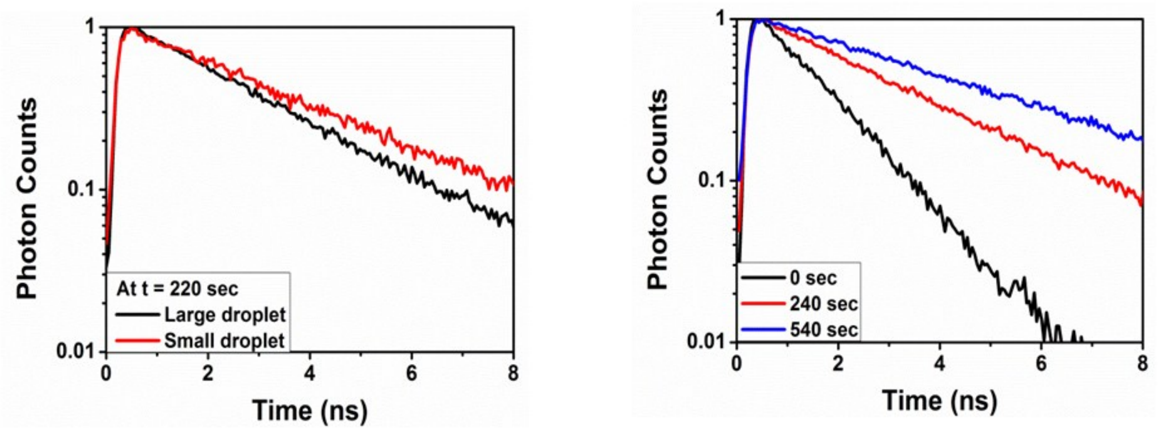

Fig. S6: Time-dependent FLIM imaging of oleic acid droplets upon exposure to 377 ppm ozone. (a) Lifetime decays recorded from the largest droplet in Figure 3a (black line) and a small droplet (red line) at t = 220 sec. (b) Lifetime decays recorded from the largest droplet at t = 0 (black), 240 (red), 540 (blue) sec. All decays can be fitted with a monoexponential model ($0.8<\chi_r^2<1.2$). All fluorescence decays of BODIPY-C_{10} were found to be monoexponential in every pixel of the image indicating that the molecular rotor was probing a micro-environment of a uniform viscosity. The decay time was shown to depend both on a particle size (a) and on the exposure time (b).

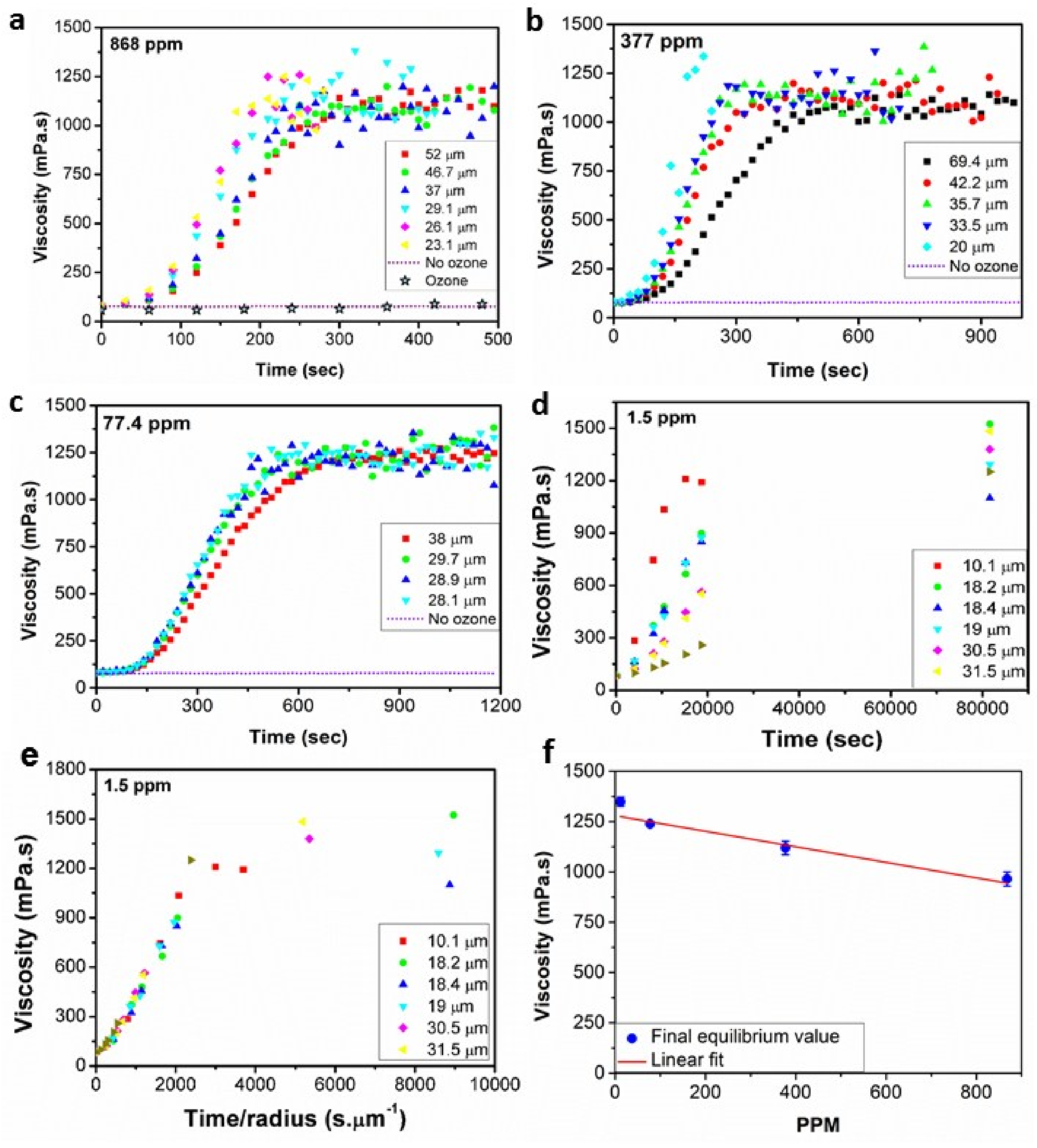

Fig. S7: Kinetic response of oleic acid droplets of varying size to decreasing concentration of ozone. (a) 868 ppm. (b) 377 ppm. (c) 77.4 ppm. (d) 1.5 ppm. The kinetic curves thus obtained, all adopt a sigmoidal shape. As the ozone concentration decreases (a to d), the rate of reaction as judged by the slope of each curve and the time taken for a reaction to complete (to achieve the maximum viscosity value) significantly slows down. At lower ozone concentrations the rate of reaction showed a distinct size dependent response, (Fig 3c main text). Importantly, two negative controls were completed to test the imaging protocol and molecular rotor. We confirmed that without ozone exposure the droplet viscosity did not change (purple dotted line, a-c) and that squalane aerosols (unreactive to ozone)

with BODIPY-C_{10} under ozone also showed no change to viscosity (turquois star black outline, FigS7a). (e) The kinetics scale with 1/radius dependence demonstrated from Fig. S7d. (f) Effect of ozone concentration (in ppm) on the final viscosity of oleic acid droplets following ozonolysis. This data shows that ozone concentration affect the final viscosity value reached upon oxidation. The final viscosity attained increases with decreasing ozone concentration, from 965±35 mPa.s for 868 ppm to 1350±24 mPa.s for 12.5 ppm. This suggests that higher ozone concentrations might impede the secondary chemical reactions, possibly reducing the opportunity for higher molecular weight products to form.

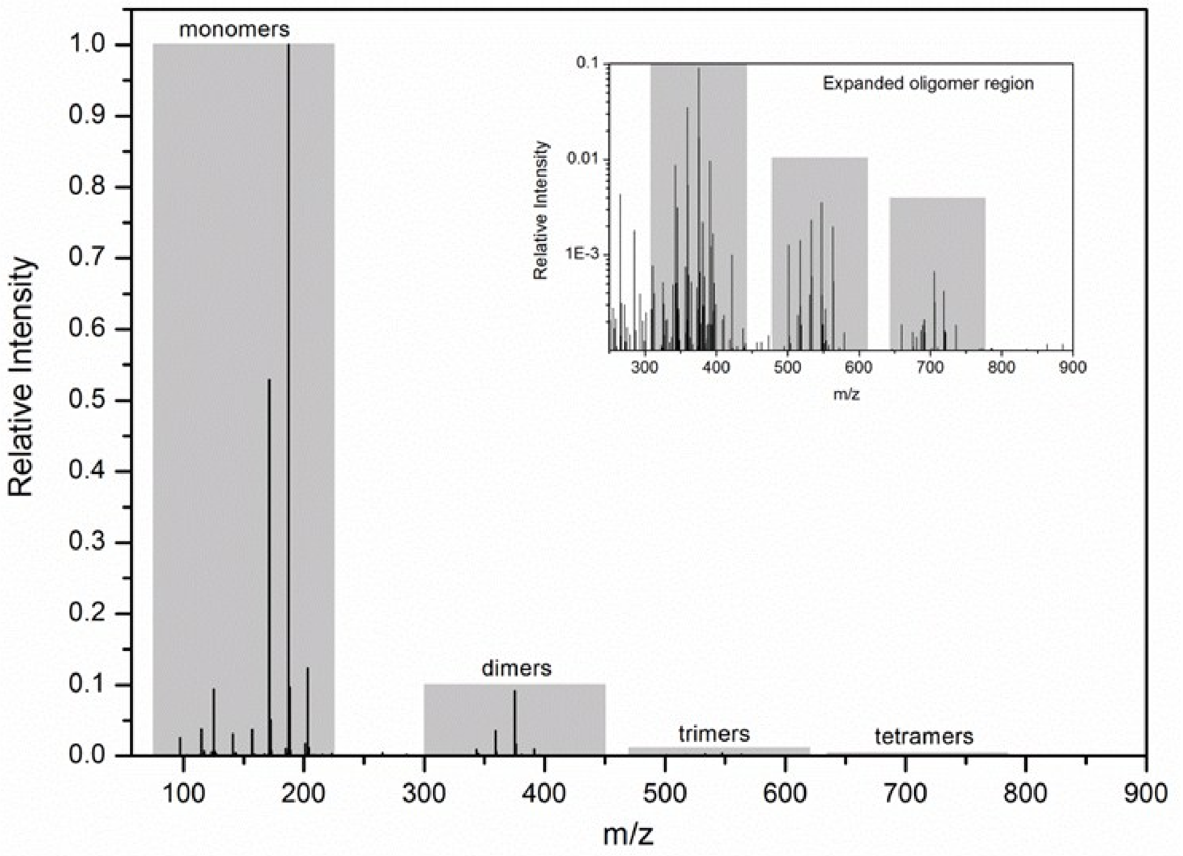

Fig. S8: Mass spectrum of oleic acid oxidation products with clearly visible monomers, dimers, trimers and tetramers including insert of expanded oligomer region. Shaded bars represent the modelled relative concentration of these groups of compounds (see also Fig. S9). A clear separation in monomeric oxidation products (most prominently the well-established known products azeleic acid, nonanoic acid and 9-oxo-nonanoic acid [2]), dimers, trimers and tetramers is visible.

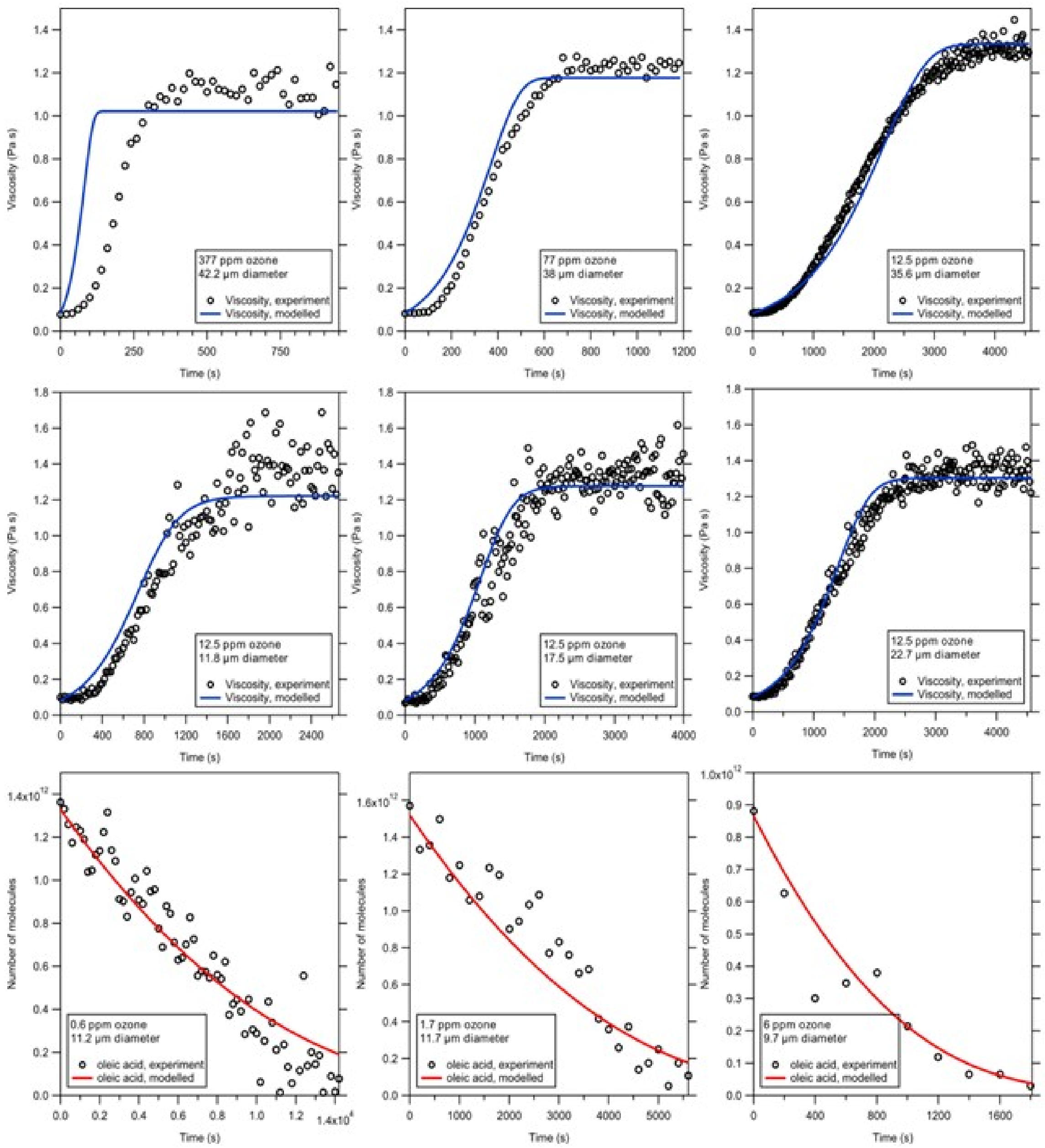

Fig. S9: Comparison of experimental data with KM-SUB model results. The modelled curves (colour lines) use a single set of kinetic parameters obtained by global optimisation (Table S1). The data (°°°) includes viscosity measurements using molecular rotors obtained in this study for a range of ozone concentrations (77-377 ppm) and a range of particles sizes; the fit for the viscosity data is shown in blue. Bottom row shows oleic acid decay measurements for three different ozone concentrations (Dennis-Smither et al. 2012 [8]); model results are shown in red. Experimental conditions (ozone concentration, particle size) are given in each respective panel.

Table S1. Parameters for KM-SUB, obtained by global optimisation to multiple experimental data sets. Kinetic parameters with square brackets around their values were fixed, all other values are fitted during the optimisation process.

Parameter	Value	Description	Dimension
$k_{BR,1}$	1.13×10^{-18}	bulk reaction rate coefficient R1	$cm^3\ s^{-1}$
$k_{BR,2}$	1.44	bulk reaction rate coefficient R2	s^{-1}
$k_{BR,3}$	5.06×10^{-18}	bulk reaction rate coefficient R3	$cm^3\ s^{-1}$
$k_{BR,4}$	1.86×10^{-17}	bulk reaction rate coefficient R4	$cm^3\ s^{-1}$
$k_{BR,5}$	1.99×10^{-16}	bulk reaction rate coefficient R5	$cm^3\ s^{-1}$
c	0.454	stoichiometric coefficient	
$H_{cp,O3}$	6.66×10^{-4}	Henry's law coefficient of O_3 in organics	$mol\ cm^{-1}\ atm$
$\tau_{d,O3}$	1.69×10^{-7}	surface desorption life time of O_3	s
$\alpha_{s,0}$	0.13	surface accommodation coefficient of O_3	
$D_{b,oligomer}$	1.19×10^{-9}	bulk diffusion coefficient of oligomers	$cm^2\ s^{-1}$
v_{dimer}	6.40	viscosity of hydroperoxide dimers	Pa s
f_{visc}	3.96	power law scaling factor for oligomer viscosity	
f_{slr}	4.41×10^{6}	scaling factor for surface reaction rate	cm^{-1}
v_{O3}	[0]	viscosity of ozone	Pa s
v_{oleic}	[0.083]	viscosity of oleic acid	Pa s
$v_{monomer}$	[0.083]	viscosity of monomeric products (NN, C9, CI)	Pa s
$D_{b, O3}$	[1.00×10^{-5}]	bulk diffusion coefficient of ozone	$cm^2\ s^{-1}$
$D_{b,oleic}$	[1.90×10^{-7}]	bulk diffusion coefficient of oleic acid	$cm^2\ s^{-1}$
$D_{b,monomer}$	[1.00×10^{-7}]	bulk diffusion coefficient of monomeric products (NN, C9, CI)	$cm^2\ s^{-1}$

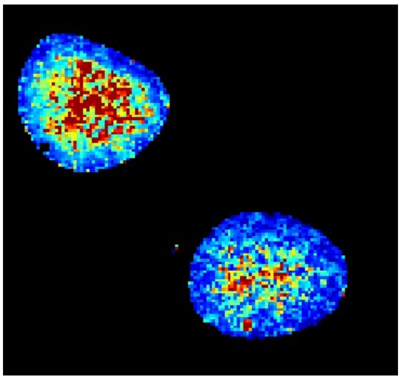

Movie S1. Visualising the diffusion fronts during hydration of oxidised myrcene aerosol (impactor collection method) by FLIM. The timescale of the movie is 0 – 23h. A selection of stills of the movie is shown in Fig. 2, main text.

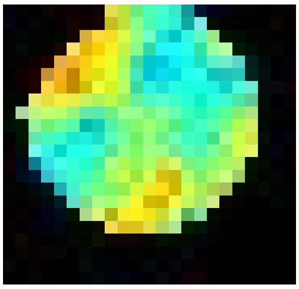

Movie S2. Visualising the development of heterogeneity in oleic acid droplet (69.4 μm diameter) during its exposure to 377 ppm of ozone for 1000 sec. The lifetime distribution in a droplet is visualised against a rainbow colour hue of 1 ns width (same increment), such that the increasing spread in colours indicates increasing heterogeneity. Note, the centre point of each 'colour hue' shifts with time as shown in the histogram of Fig. 4b, main text. A selection of stills from the movie is shown in Fig. 4b, main text.

References

1 M. Shiraiwa, C. Pfrang and U. Pöschl, *Atmos. Chem. Phys.*, 2010, **10**, 3673–3691.

2 O. Vesna, M. Sax, M. Kalberer, A. Gaschen and M. Ammann, *Atmos. Environ.*, 2009, **43**, 3662–3669.

3 P. J. Ziemann, *Faraday Discuss.*, 2005, **130**, 469.

4 J. W. L. Lee, V. Carrascón, P. J. Gallimore, S. J. Fuller, A. Björkegren, D. R. Spring, F. D. Pope and M. Kalberer, *Phys. Chem. Chem. Phys.*, 2012, **14**, 8023–31.

5 D. S. Viswanath, T. K. Ghosh, D. H. L. Prasad, N. V. K. Dutt and K. Y. Rani, *Viscosity of Liquids: Theory, Estimation, Experiment, and Data [Hardcover]*, Springer; 2007 edition, Dordrecht, 2006.

6 H. Noureddini, B. C. Teoh and L. Davis Clements, *J. Am. Oil Chem. Soc.*, 1992, **69**, 1189–1191.

7 D. R. Hanson, A. R. Ravishankara and S. Solomon, *J. Geophys. Res.*, 1994, **99**, 3615.

8 B. J. Dennis-Smither, K. L. Hanford, N.-O. A. Kwamena, R. E. H. Miles and J. P. Reid, *J. Phys. Chem. A*, 2012, **116**, 6159–68.

9 T. Berkemeier, A. J. Huisman, M. Ammann, M. Shiraiwa, T. Koop and U. Pöschl, *Atmos. Chem. Phys.*, 2013, **13**, 6663–6686.

10 G. D. Smith, E. Woods, C. L. DeForest, T. Baer and R. E. Miller, *J. Phys. Chem. A*, 2002, **106**, 8085–8095.

11 N. A. Hosny, G. Mohamedi, P. Rademeyer, J. Owen, Y. Wu, M.-X. Tang, R. J. Eckersley, E. Stride and M. K. Kuimova, *Proc. Natl. Acad. Sci. U. S. A.*, 2013, **110**, 9225–30.
